# MiR-328 Expression Is Decreased in High-Grade Gliomas and Is Associated with Worse Survival in Primary Glioblastoma

**DOI:** 10.1371/journal.pone.0047270

**Published:** 2012-10-12

**Authors:** Zhifeng Wu, Lihua Sun, Hongjun Wang, Jianshe Yao, Chuanlu Jiang, Wenhui Xu, Zhengxiang Yang

**Affiliations:** 1 Department of Neurosurgery, the Affiliated Wuxi People’s Hospital of Nanjing Medical University, Wuxi, People’s Republic of China; 2 Department of Neurosurgery, the Second Affiliated Hospital of Harbin Medical University, Harbin, People’s Republic of China; 3 Department of Neurosurgery, the Affiliated Yixin People’s Hospital of Jiangsu University, Yixin, People’s Republic of China; Beijing Tiantan Hospital, Capital Medical University, China

## Abstract

MicroRNAs, a group of small endogenous, noncoding RNAs, are aberrantly expressed in many human cancers and can act as oncogene or anti-oncogene. Recent evidence suggests that some miRNAs have prognostic value for tumors. MiR-328 is known as a tumor suppressor; however, its relationship with the clinicopathological features of glioblastoma (GBM) and its prognostic value has yet not been investigated. We found that expression of miR-328 was significantly decreased both in anaplastic and GBM cohorts and that low miR-328 expression also conferred poor survival in primary GBM (PGBM) patients. MiR-328 might, therefore, serve as an independent prognostic marker. Furthermore, expression profiles of miR-328-associated mRNAs were established via microarrays for 60 GBM samples. The ontology of the miR-328-associated genes was then analyzed, which identified gene sets tightly related to cell mitosis. In addition, ectopic expression of miR-328 inhibited U87 cell proliferation and induced U87 cell cycle arrest. In conclusion, this is the first report showing that miR-328 is associated with patient’s survival time and that miR-328 might serve as an independent prognostic biomarker for GBM.

## Introduction

Glioma is the most common neoplasm of the central nervous system (CNS), with glioblastoma (GBM) being the most malignant type [1]. Despite advances in treatment, the median survival of newly diagnosed GBM patients is only 9–14.6 months [2], [3] and GBM remains refractory to conventional therapies. Interestingly, the survival time of GBM patients ranges from one week to over 5 years following diagnosis [4], [5], which may reflect mutation or altered expression levels of key genes. Over recent years, more and more molecular markers for GBM have been identified, many of which are now used for assessment and management of GBM. For example, O-6-methylguanine-DNA methyltransferase (MGMT) promoter methylation is used to predict the response to temozolomide (TMZ) therapy [6], [7], and isocitrate dehydrogenase 1 *(IDH1*) can be used for prognosis [8]. However, no biomarker is precise enough to enable individual assessment of individual GBM patients.

MicroRNAs (MiRNAs) are small endogenous noncoding RNAs that modulate the expression of multiple target genes at the post-transcriptional level by binding with target mRNA sequences in the 3′untranslated region (UTR) [9]. Abnormal expressions of miRNAs is linked with the origin and development of tumors and miRNAs can act as either activators or suppressors [10], [11], [12]. Furthermore, the use of miRNAs as diagnostic, prognostic or/and therapeutic response markers in tumors has received great interest in the last few years [13], [14], and specific miRNA expression signatures can indicate the diagnosis, therapy response and prognosis of cancers [15], [16], [17]. It has been reported that the expression of some miRNAs is associated with outcome for GBM patients [18], [19]. However, no stable prognostic miRNA has been validated for GBM. Given that miRNAs can exist in tumor samples without degradation for a long time [20], it would be valuable to identify predictive miRNAs in GBM.

MiR-328, a tumor suppressor, is involved in the aggressive progression of gliomas, which suggests that it may play an important role in the malignant transformation of gliomas [21]. However, the expression level of miR-328 and its relationship with pathological features and overall survival in human GBM is still unclear. Here, we report that the expression of miR-328 was decreased in GBM samples and in anaplastic gliomas compared with low grade gliomas by using the expression values from microarrays of 198 frozen glioma tissues. These results were confirmed in 100 independent samples. And overall survival analysis on primary GBM (pGBM) patients showed that patients with lower than median levels of miR-328 expression were associated with decreased survival time relative to those with miR-328 expression levels higher than the median. Furthermore, multivariate Cox proportional hazards analysis showed that miR-328 could act as an independent prognostic biomarker. Integrated analysis of miR-328 with whole genome gene expression patterns showed that low-expression of miR-328 was tightly associated with the gene sets related to cell cycle. In addition, functional assays showed that miR-328 might serve as a potential anti-proliferation target for GBM therapy.

**Figure 1:**
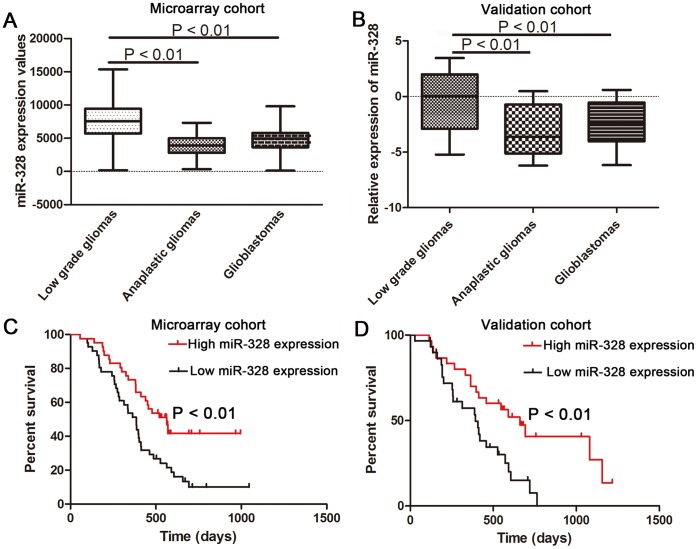
MiR-328 expression pattern in glioma samples and its association with overall survival time in GBMs. (A) Expression values of miR-328 in the microarray cohort containing 198 frozen glioma tissues. (B) Relative expression levels of miR-328 in the validation cohort containing 100 glioma tissues. (C) MiR-328 expression values in 82 frozen primary GBM tissues were subjected to Kaplan-Meier survival analyses. (D) Kaplan-Meier survival curves according to the relative expression levels of miR-328 in an independent 60 GBM tissues. The log-rank test was used to calculate p values.

## Materials and Methods

### Samples and Patients

Total 100 glioma samples were used in the “Validation” cohort (20 low grade gliomas, 20 anaplastic gliomas and 60 GBMs). All the samples were confirmed by pathological diagnosis according to the 2007 WHO classification. After resection, all samples were immediately frozen in liquid nitrogen until RNA extraction. Extent of resection was graded as gross total resection (GTR) or non-GTR using MRIs obtained within 72 h after surgical resection by two independent radiologists. And GBM samples with *IDH1* mutation were detected as previously report [8]. This study was approved by the institutional review boards of Nanjing Medical University and the Second Affiliated Hospital of Harbin Medical University, and written informed consent was obtained from all patients.

**Table 1:** Multivariable Cox proportional hazard regression analyses of miR-328 expression and clinicopathologic characteristics in relation to overall survival in patients with pGBM.

Variable	HR	95% CI	p Value
KPS score			
≤80	1.00		
>80	0.54	0.27–1.07	0.08
Extent of resection			
Subtotal	1.00		
Total	0.5	0.26–0.99	<0.05
miR-328 expression			
Low	1.00		
High	0.46	0.26–0.82	<0.01
*IDH1* mutation status			
No mutation	1.00		
Mutation	0.62	0.24–1.6	0.32

HR, hazard ratio.

### Cell Culture and Oligonucleotide Transfection

Human glioma cell line U87 were purchased from the Chinese Academy of Sciences Cell Bank, all cell lines were maintained in a 37°C, 5% CO_2_ incubator in DMEM supplemented with 10% fetal bovine serum (FBS). 2′-O-methyl (2′-O-Me) hsa-miR-328 mimic (miR-328 sense oligonucleotide) and miR-328 negative control (NC) were chemically synthesized by Shanghai GenePharma Company (Shanghai, China). The miR-328 mimic sequence is 5′-CUG GCC CUC UCU GCC CUU CCG U-3′, 5′-GGA AGG GCA GAG AGG GCC AGU U-3′, and the NC sequence is 5′-UUC UCC GAA CGU GUC ACG UTT-3′, 5′-ACG UGA CAC GUU CGG AGA ATT-3′. Cells of 70–80% confluence were transfected with oligonucleotide using Lipofectamine 2000 reagent (Invitrogen, Carlsbad, USA). Transfection was performed according to the manufacturer’s instructions, and the final oligonucleotide concentration was 10 nmol/L. Medium was replaced 6 h after transfection [12].

**Figure 2:**
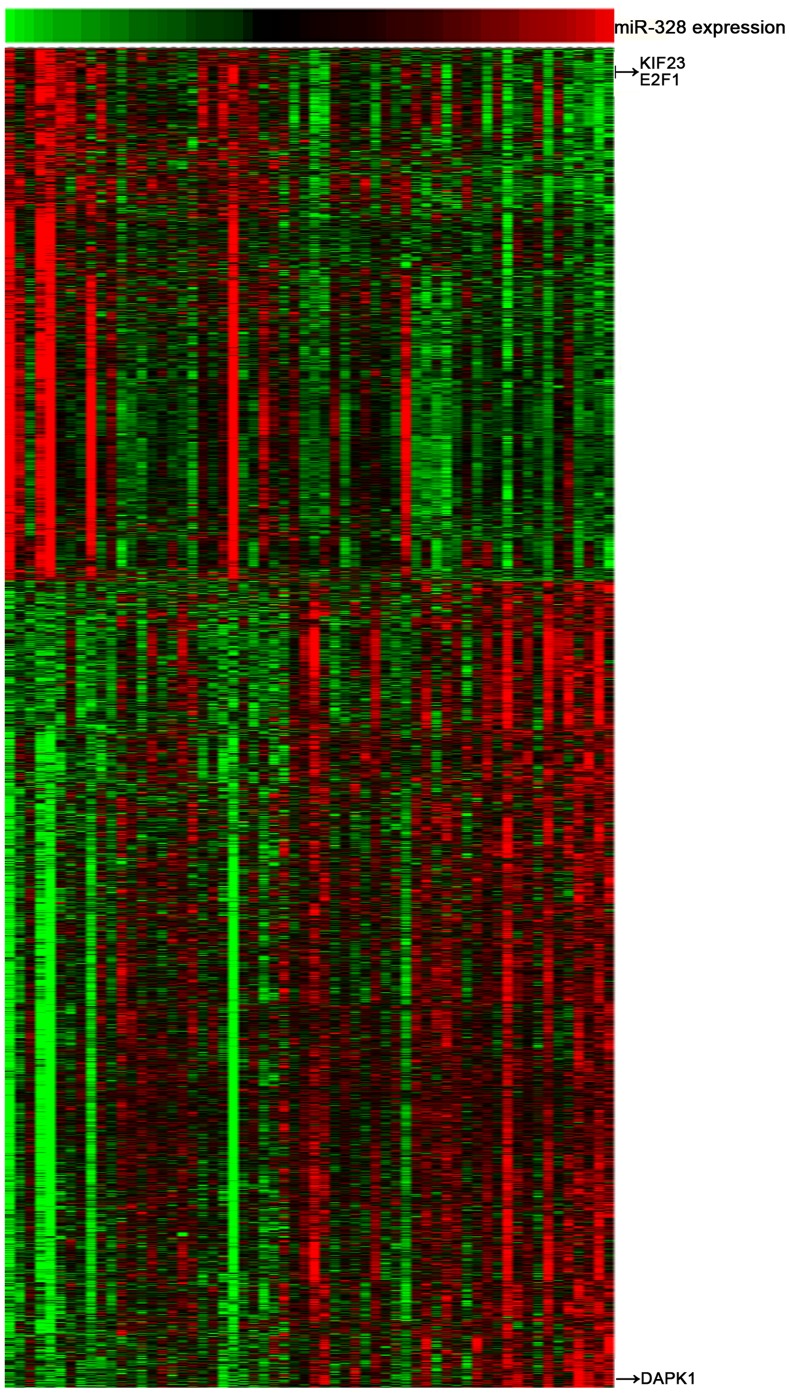
Heat map of the gene-expression signature correlated with miR-328 expression. Columns represented patients and rows represented probe sets. Patients were arranged from left to right by increasing levels of miR-328 expression. Expression levels of the probe sets were represented by color, with green demonstrating expression lower and red demonstrating expression higher than the median value of the given probe set. Arrows indicated the genes which were discussed in the article.

**Table 2:** The biologic processes associated with miR-328 low expression in pGBMs.

Name	P value
**Regulation of transcription**	3.1E-11
**transcription**	6.5E-11
**Cell cycle process**	1.4E-9
**Cell cycle phase**	1.9E-9
**M phase**	2.1E-9
**M phase of mitotic cell cycle**	5.9E-9
**Cell division**	7.6E-9
**Nuclear division**	1.3E-8
**Mitosis**	1.3E-8
**Organelle fission**	3.6E-8
**Cell cycle**	4.5E-8

### RNA Extraction

Total RNA was extracted from frozen tissues by using mirVana miRNA Isolation kit (Ambion, Austin, TX, USA) according to the manufacturer’s protocol. RNA concentration and quality were measured using the NanoDrop ND-1000 spectrophotometer (NanoDrop Technologies, Houston, TX, USA) and then stored at −80°C until use.

**Figure 3:**
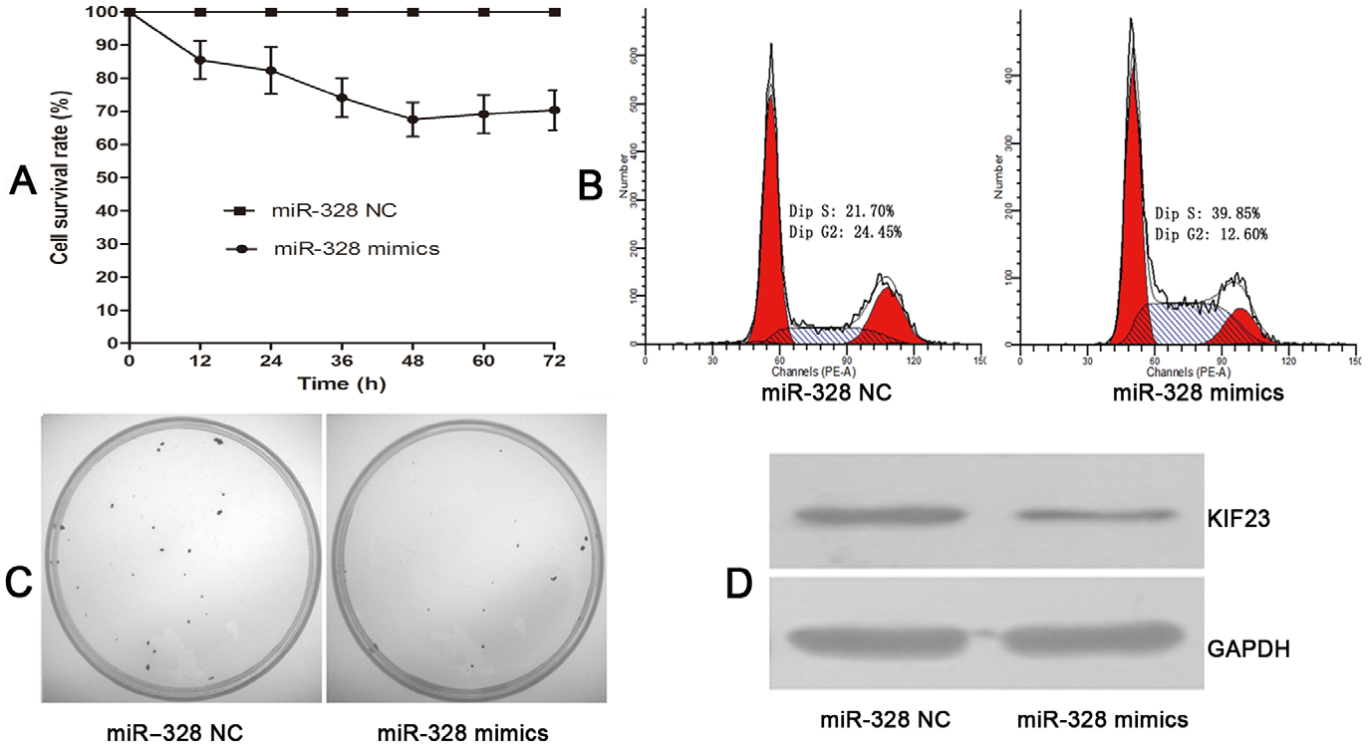
MiR-328 suppresses U87MG cell proliferation *in vitro*. (A) MTT assay showed that U87 cell growth was significantly inhibited by miR-328 mimics. (B) Cell cycle analysis demonstrated that ectopic expression of miR-328 caused U87 MG cell S-phase arrest. (C) Clonogenic assay indicated that miR-328 mimics could suppress U87MG cell clone formation. (D) KIF23 protein was measured by western blot, and the result showed that miR-328 can modulate KIF23 expression.

**Figure 4:**
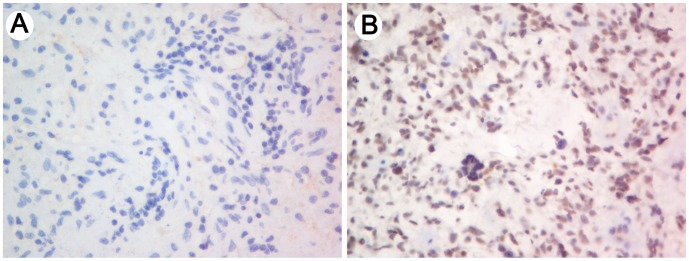
KIF23 protein levels in pGBM samples. The KIF23 staining score  =  staining intensity × proportion of positively stained tumor cells. (A) Low KIF23 expression was defined as a staining score ≤4; (B) High KIF23 expression was defined as a staining score >4.

**Table 3:** MiR-328 expression values showed negative correlation to KIF23 protein expression levels in 60 pGBM samples.

P<0.05	Low KIF23	High KIF23
Low miR-328	9	21
High miR-328	18	12

### MiR-328 Expression Analysis

MiR-328 expression values of 198 “Microarray” samples were from Chinese Glioma Genome Atlas (CGGA) (among the 198 “Microarray” samples, 63 samples were diagnosed as low grade gliomas, 44 were anaplastic gliomas and 91 were GBMs). And the levels of miR-328 expression in the “Validation” glioma cohort were detected by TaqMan-based real-time quantification PCR (qRT-PCR). The primers and probes of has-miR-328 and U6B small nuclear RNA gene (RUN6B) used as endogenous control for TaqMan miRNA assays were purchased from Applied Biosystems, qRT-PCR was performed according to the manufacturer’s instructions on the ABI 7300 HT Sequence Detection System (Applied Biosystems, CA). The relative level of miR-328 was calculated by using 2^−△△Ct^ method.

### Gene Ontology Analysis

The correlation analysis of miR-328 and whole genome gene expression was performed in 60 pGBM samples with paired miRNA and mRNA profiling (CGGA). To investigate the biological processes that correlate with miR-328 expression in pGBM, miR-328 associated genes were projected to Gene ontology analysis (http://david.abcc.ncifcrf.gov).

### Immunohistochemistry (IHC)

Immunohistochemical staining with streptavidin-biotin immunoperoxidase assay was performed on forty formalin-fixed, paraffin-embedded pGBM tissues to detect KIF23 expression by using rabbit-anti KIF23 (Abcom, 1∶200) primary antibody. Slides were individually reviewed and scored by two independent observers. The proportion of positively-stained tumor cells was graded as follows: 0 (no positive tumor cells); 1 (<10% positive tumor cells); 2 (10–30% positive tumor cells); and 3 (>30% positive tumor cells). The intensity of staining was graded as follows: 0 (no staining), 1 (weak staining, light yellow), 2 (moderate staining, yellowish brown), and 3 (strong staining, brown). The staining score  =  staining intensity × proportion of positively stained tumor cells. High KIF23 expression was defined as a staining score >4, while low expression was defined as a staining score ≤4. The final immunostaining score was the average of the two observers.

### 3-(4, 5-Dimethylthiazol-2-yl)-2, 5-diphenyltetrazolium Bromide (MTT) Assay

The MTT assay was used to determine the cell viability. Cells (1×10^4^) were plated in 96-well plates with six replicate wells for each condition, after each treatment at 12 h, 24 h, 36 h, 48 h, 72 h, MTT (Sigma, USA) assay was done, the cell viability was determined at 540 nm absorbance using a scanning multiwell spectrophotometer (enzyme-linked immunosorbent assay plate reader, Biotek Instruments Inc.). All data points represent the mean of a minimum of six wells.

### Clonogenic Assay

Cells in the log phase of growth were digested with 0.25% pancreatin. Cells were then dissociated and a single cell suspension was obtained. 50 cells were seeded onto a culture plate, after adherence, cells were transfected with miR-328 mimics or a negative control. Six hours later medium was replaced. One week later, oligonucleotide transfection was repeated. Plates were incubated in a 37°C, 5% CO_2_ incubator for 2 weeks and cell colonies were photographed after being stained with crystal violet.

### Cell Cycle Analysis

After treatment with miR-328 mimics for 48 h, cell cycle analysis was applied, in brief, cells were collected, washed with phosphate buffered saline (PBS) and then fixed with 75% ethanol at −20°C for 1.5 h, then cells were suspended in hanks balanced salt solutions (HBSS) containing 50 µg/ml of RNaseA (Boehringer Mannheim, Indianapolis, IN) and 50 µg/ml of propidium iodide (PI) (Sigma-Aldrich), incubated for 1 h at room temperature and then analyzed by FACScan (Becton Dickinson, San Jose, CA).

### Western Blotting Assay

Total proteins were collected by using Total Protein Extraction Kit (KeyGen, China), 30 ug protein were added for One-dimensional sodium dodecyl sulfate (SDS)-polyacrylamide gel electrophoresis using the discontinuous buffer system of Laemmli (Bio-Rad Laboratories, USA). The electrophoresed proteins were transferred to a polyvinylidene difluoride membrane and subjected to immunoblot analysis with rabbit-anti KIF23 (Abcom, 1∶800), glyceraldehyde-3-phosphate dehydrogenase (GAPDH) was used for control.

### Statistical Analysis

Statistical analysis was performed using SPSS Graduate Pack 13.0, Metlab2009a and GraphPad Prism 5.0 statistical software. Descriptive statistics include means ± SE. Student’s t test, one-way ANOVAs and Exact Sig (2-sided) χ^2^ test were used to analyze significant differences. Kaplan-Meier survival analysis was used to estimate the survival distributions, and the log-rank test was used to assess the statistical significance between stratified survival groups. Karnofsky performance status (KPS), extent of resection, and IDH1 mutation were incorporated into a Cox multivariate analysis. A two-sided P value of <0.05 was considered significant.

## Results

### Expression of miR-328 is Decreased in GBM Tissues and Confers a Poor Prognosis

To detect the expression pattern of miR-328 in glioma samples, a microarray of 198 glioma samples (63 low-grade gliomas, 44 anaplastic gliomas and 91 GBMs) was analyzed, just as shown in Fig.1A. GBMs and anaplastic gliomas demonstrated a significantly lower levels of miR-328 expression compared with low-grade gliomas (p<0.01). To further confirm this result, qRT-PCR was performed to measure miR-328 levels in an independent cohort containing 100 glioma samples (20 low-grade gliomas, 20 anaplastic gliomas and 60 GBMs). As shown in Fig. 1B, miR-328 was significantly down-regulated in GBMs and anaplastic gliomas compared with low grade gliomas. Furthermore, the relationship between miR-328 expression and overall survival was determined through Kaplan-Meier survival curve analysis with a log-rank comparison of 82 pGBM patients. In the microarray cohort, pGBM patients with lower than median levels of miR-328 expression had poorer survival in contrast to those with higher than median levels of miR-328 expression (Fig. 1C) who had longer survival times. Furthermore, this correlation of miR-328 expression with overall survival was also observed in the independent validation samples (p<0.01) (Fig. 1D).

### MiR-328 is an Independent Prognostic Biomarker for GBM

To determine the prognostic value of miR-328 in GBM, we first employed a univariate Cox comparison. High levels of miR-328 expression was a “protective” factor in GBM (p<0.01, HR = 0.46, 95%CI = 0.27–0.79). Next, we performed multivariate Cox proportional hazards analysis incorporating miR-328 expression, KPS, *IDH1* mutation and extent of resection. This analysis revealed that miR-328 expression and extent of resection were significantly correlated with survival (Table 1). These results indicated that miR-328 could be used as an independent prognostic biomarker for GBM.

### MiR-328 is Tightly Correlated to the Cell Cycle Process

To investigate the cell biological processes associated with miR-328 expression in pGBM, the integrated analysis of miR-328 expression levels and whole genome gene profiling were performed in 60 pGBM samples. 1625 probes were positively correlated (R>0.4, p<0.01) and 1072 probes were negatively correlated (R<-0.4, p<0.01) to miR-328 levels (Fig. 2). Gene ontology analysis suggested that miR-328 was significantly associated with the gene sets related to cell cycle. By using a p value<0.001, processes mainly related to transcription, the cell cycle processes of M phase, mitosis, cell division, nuclear division and mitosis were significantly enriched in the pGBM samples where miR-328 was down-regulated (Table 2). This analysis indicates that miR-328 might have essential roles in glioma cells proliferation.

### Ectopic miR-328 Expression Suppresses Cell Proliferation in GBM

To study the functional role of miR-328 in GBM cell proliferation, miR-328 mimics were transfected into U87MG cells to elevate miR-328 expression levels, while miR-328 NC was used as a negative control. Cell viability was measured using the MTT assay and the clonogenic assay. Cells were transfected with miR-328 mimics or NC and 12 h to 72 h later were subjected to the MTT assay. As shown in Fig. 3, cell growth was significantly suppressed by miR-328 mimics and the maximum inhibition rate was 48 h after transfection. The clonogenic assay showed that miR-328 mimics significantly inhibited the formation of cell clones. Kinesin family member 23 (KIF23) is a nuclear protein that localizes to the interzone of mitotic spindles and acts as a plus-end-directed motor enzyme that moves anti-parallel microtubules [22]. KIF23 is essential for cellular shape and processes such as motility, mitosis, intracellular vesicle transport [23], [24]. In glioma, down-regulation of KIF23 could suppress glioma proliferation both *in vivo* and *in vitro*, and glioma patients with high levels of KIF23 expression also tend to show poorer prognosis compared with patients with low KIF23 expression [25]. In our study, KIF23 was found to be inversely related to miR-328 expression by whole geneome gene profiling, and functional studies showed that miR-328 mimics could suppress KIF23 protein levels, furthermore, KIF23 protein expression levels showed negative correlation to miR-328 expression values (Fig. 3&4, Table 3). Together, these results suggest that miR-328 could be an anti-proliferation target for GBM treatment.

## Discussion

Recently, more and more miRNAs have been found to have diagnostic, prognostic and/or therapeutic prediction values in cancers [13], [26]. And in glioma, miR-182 was reported to have prognostic values both in low grade gliomas and high grade gliomas [27]. MiR-196 was found to be related to overall survival, despite the fact that two studies found opposite conclusions [28], [29]. And miR-181b, miR-106a, and miR-21 were significantly associated with patients’ survival time in astrocytoma [30]. However, no miRNA with significantly prognostic value has been reported based on larger GBM samples.

MiR-328 is expressed at low levels in many cancers. It has been reported that miR-328 can decrease the chemoresistance of GBM stem cells by down-regulating ABCG2 protein levels [31]. Furthermore, in the malignant progression of glioma (primary WHO grade II gliomas spontaneously progress to WHO grade IV secondary GBMs), miR-328 shows reduced expression upon progression [21]. Together, this evidence suggests that miR-328 has an essential role in the original and/or progression of GBM. Thus, it is valuable to further confirm the functional role and predictive value of miR-328 expression in human GBM. In this study, we first evaluated the expression levels of miR-328 in 198 frozen glioma samples using microarray technology. Furthermore, an independent cohort containing 100 glioma samples were used to validate the expression pattern of miR-328 in gliomas. Both of the cohorts showed that miR-328 expression was significantly decreased in GBMs and anaplastic gliomas relative to low-grade gliomas. Kaplan-Meier survival analysis of 82 frozen pGBM samples revealed that low expression levels of miR-328 conferred a poor prognosis. The prognostic value of miR-328 was also validated in the independent GBM samples. Furthermore, to confirm whether miR-328 could be served as an independent prognostic biomarker in GBMs, miR-328 expression was analyzed by univariate Cox regression analysis. *IDH1* mutation, KPS, extent of resection and miR-328 expression were subjected to multivariate Cox proportional hazards analysis, which showed that miR-328 could be used as an independent prognostic biomarker for GBM. In addition, to explore the biological processes of miR-328 in GBMs. 60 pGBM samples with variable miR-328 transcript levels were analyzed by whole genome gene profiling. We observed a positive correlation of miR-328 expression with a series of tumor suppressors which mediate cell cycle arrest, including death-associated protein kinase 1 (DAPK1) [32]. Furthermore, oncogenes which drive cell proliferation and cell mitosis, including KIF23 [25] and E2F transcription factor 1 (E2F1) [33], were found to negatively correlate with the expression of miR-328. In addition, the correlation analysis of KIF23 protein levels by IHC and miR-328 expression by qRT-PCR were performed on 60 pGBM tissues. Further, a negative correlation was found. *In vitro* functional analysis showed that ectopic miR-328 expression significantly suppressed U87MG cells proliferation. These results suggest that miR-328 might affect malignant progression and function as an anti-oncogene via the suppressing of GBM cell proliferation.

In conclusion, our studies show that miR-328 expression is decreased in GBMs. GBM patients with lower than median levels of miR-328 expression have poor survival. We also show that miR-328 is closely related to cell cycle progression; ectopic miR-328 expression in GBM cells could significantly suppress cell proliferation. Together, these results indicate that miR-328 is a tumor suppressor and could be a potential target for anti-proliferative therapy. In addition, miR-328 might be used as an independent prognostic biomarker for GBM.
